# Clinical and Laboratory Characteristics for the Diagnosis of Bacterial Ventriculitis After Aneurysmal Subarachnoid Hemorrhage

**DOI:** 10.1007/s12028-016-0345-8

**Published:** 2016-12-21

**Authors:** J. Hoogmoed, D. van de Beek, B. A. Coert, J. Horn, W. P. Vandertop, D. Verbaan

**Affiliations:** 1 Department of Neurosurgery, Neurosurgical Center Amsterdam, Academic Medical Center, Meibergdreef 9, 1105 AZ Amsterdam, The Netherlands; 2 Department of Neurology, Academic Medical Center, Amsterdam, The Netherlands; 3 Department of Intensive Care Medicine, Academic Medical Center, Amsterdam, The Netherlands

**Keywords:** Cerebral ventriculitis, Infectious ventriculitis, Meningitis, Bacterial, Subarachnoid hemorrhage, Drainage, Neurosurgery, Anti-bacterial agents, Intensive care units

## Abstract

**Background:**

The diagnosis of nosocomial bacterial ventriculitis in patients with subarachnoid hemorrhage (SAH) can be challenging.

**Methods:**

We performed a retrospective study on the diagnostic accuracy of clinical and laboratory characteristics for the diagnosis of bacterial ventriculitis in 209 consecutive patients with an aneurysmal SAH admitted in a tertiary referral center from 2008 to 2010. Diagnostic value of clinical characteristics and inflammatory indexes in CSF and blood were determined for three diagnostic categories: (1) no suspicion for bacterial ventriculitis; (2) clinical suspicion for bacterial ventriculitis, defined as initiation of empirical antibiotic treatment for ventriculitis, but negative CSF cultures; and (3) CSF culture-positive bacterial ventriculitis.

**Results:**

Empirical antibiotics for suspected ventriculitis was initiated in 48 of 209 (23 %) patients. CSF cultures were positive in 11 (5 %) patients. Within the group of suspected ventriculitis, only longer duration of CSF drainage and lower CSF red blood cell counts predicted for culture positivity. None of the other clinical features or inflammatory indexes in CSF and blood were associated with culture-proven bacterial ventriculitis.

**Conclusions:**

Nosocomial bacterial ventriculitis in patients with aneurysmal SAH is often suspected but confirmed by culture in a minority of cases. Improvement of diagnostics for nosocomial bacterial ventriculitis in patients with aneurysmal SAH is needed.

## Introduction

Infections in patients with a subarachnoid hemorrhage (SAH), mainly pneumonia, urinary tract infections, blood stream infections, or bacterial ventriculitis, are independently associated with a prolonged stay in the intensive care unit (ICU), a poor outcome and increased mortality [1, 2]. Bacterial ventriculitis is reported in 3–29 % of SAH patients [2–6] and is strongly associated with the placement of CSF (cerebrospinal fluid) catheters [5, 7]. Although infections and fever after SAH have been studied, which includes ventriculitis, no specific study has paid attention specifically and solely to suspected bacterial ventriculitis in this subgroup [1, 3–6, 8]. In studies on external catheter-related bacterial ventriculitis, subgroup analyses specifically for SAH patients have not been performed [9–16]. Although the incidence of external catheter-related bacterial ventriculitis is not expected to be different in other brain injury patients, the clinical symptoms of SAH closely resemble those of bacterial ventriculitis and can thus influence the clinical diagnosis of a bacterial ventriculitis.

A clinical suspicion of nosocomial bacterial ventriculitis should prompt a diagnostic workup and antimicrobial therapy [7]. The diagnostic workup in these patients typically consists of neuroimaging, cerebrospinal fluid analysis (cell counts, Gram’s staining, cultures, biochemical tests for glucose and protein), and cultures of blood. The interpretation of the numbers of white cells in cerebrospinal fluid is especially problematic in patients who have bacterial ventriculitis that develops after intraventricular and subarachnoid hemorrhage; although a formula has been proposed for interpretation [13], the diagnostic accuracy is unknown. The diagnosis of nosocomial bacterial ventriculitis is made on the basis of the results of a cerebrospinal fluid culture, of which the results can be false negative. We performed a retrospective cohort study to investigate the diagnostic accuracy of clinical and laboratory characteristics in bacterial ventriculitis after SAH.

## Materials and Methods

### Participants

The Academic Medical Center (AMC) in Amsterdam (The Netherlands) acts as a tertiary referral center for patients with an aneurysmal SAH in a region of approximately 1.3 million people. Consecutive patients admitted with an aneurysmal SAH were included in this study. These patients were collected from a prospectively kept database of all SAH patients admitted in the AMC. There was no objection of the Medical Ethics Committee of the AMC to perform this study. Patients were excluded if there was no clinical data available (for example due to early referral to another hospital). Clinical records, from the electronic patient chart, were retrospectively examined. World Federation of Neurosurgical Societies (WFNS) grade, Fisher grade, and length of stay were documented. WFNS grade is a five category (I–V) neurological grading scale based upon the Glasgow Coma Score (GCS) in which a higher score indicates a worse clinical condition [17]. Grades were categorized as a dichotomous variable: good (I–III) and poor (IV–V) grade. The Fisher grade is a four-point scale for classification of the amount of extravasated blood on the initial CT scan (grade 1: no visible hematoma, grade 4: intraventricular or intracerebral hematoma) [18]. Grades were dichotomized into good (Fisher grade 1–3) and poor (Fisher grade 4) grades. Treatment procedures (i.e., coiling, surgical clipping, decompressive craniotomy), catheter placement (external ventricular, lumbar or cisternal catheter), number of catheters and duration of drainage were documented.

Placement and maintenance of the external ventricular and lumbar catheters was performed according to a standardized protocol to minimize the risk of infection [19]. External ventricular catheter placement was performed under sterile circumstances in the operating theater. Standard external ventricular catheters without antibiotic or silver impregnation were used. The external ventricular catheters were tunneled subcutaneously for at least 5 cm. External lumbar catheters were placed at bedside under sterile conditions and also tunneled subcutaneously. In most patients who underwent a craniotomy for surgical clipping of an aneurysm, a cisternal catheter was placed for postoperative CSF drainage during five days. All types of catheters were tunneled subcutaneously. In all procedures, patients received prophylactic antibiotics at least 30 min before the start of the surgery: ceftriaxone 1000 mg for an external ventricular catheter or a craniotomy, flucloxacillin 1000 mg for an external lumbar catheter. The catheters were attended to according to a standardized nursing protocol. No routine microbiological or chemical tests of CSF were taken. External ventricular or lumbar catheters were not routinely changed, they were only changed if there was a dysfunction or if there was an intractable infection.

Selective decontamination of the digestive tract was given to all patients in the ICU who were expected to be ventilator-dependent for at least two days. SDD was used as prevention of secondary colonization of patients with Gram-negative bacteria in order to reduce mortality [20]. It consisted of orabase (polymyxin E 100 mg, tobramycin 80 mg, amphotericin B 500 mg), four times daily orally and intravenous cefotaxime 3 g/24 h continuously during 4 days.

### Bacterial Ventriculitis and Diagnostic Testing

Bacterial ventriculitis was suspected in SAH patients if they experienced a (new) period of fever (see below for definition) and/or altered mental status and/or (new) nuchal rigidity. Other causes of infection were excluded by performing blood, bronchial and urinary cultures, plain chest X-rays and physical examination. Other causes of an altered mental status, like hydrocephalus or cerebral ischemia, were excluded by performing a plain CT scan.

Clinical records were evaluated to identify patients who were treated for bacterial ventriculitis. If antibiotics were started specifically for ventriculitis, this patient was defined as ‘clinically suspected bacterial ventriculitis’. All patients who were started on these antibiotics were included irrespective if they underwent transcranial surgery or had an external ventricular or lumbar catheter placed. Data from the chemical analyses of blood [C-reactive protein (mg/L), white blood cell count (10E9/L) and glucose levels (mmol/L)] and CSF [red blood cells (cells/μL), white blood cell count (cells/μL), protein (g/L) and glucose levels (mmol/L)] and microbiological analyses of CSF were collected. CSF was collected at the time of clinical suspicion of a bacterial ventriculitis from the external ventricular or lumbar catheter in place. If there was no CSF catheter in place, a lumbar puncture was performed. Bacterial ventriculitis was defined by a positive CSF culture for bacteria; however, if cultures revealed *Staphylococcus epidermidis*, two consecutive positive cultures were need to rule out contamination.

Of all patients, the daily maximum temperature (*T*_max_) was collected. Fever was identified as a *T*_max_ ≥ 38.5 °C. For the calculation of the *T*_max_ in patients with suspected bacterial ventriculitis the *T*_max_ until, and including, the day the clinical suspicion of bacterial ventriculitis rose (see definition of bacterial ventriculitis) was used. This was done in order to exclude the days after the (suspected) bacterial ventriculitis, which are not relevant for comparison. For the patients not suspected of bacterial ventriculitis, the *T*_max_ during the whole admission period was used. The number of fever days was defined as the sum of days with a *T*_max_ ≥ 38.5 °C during the total period of hospitalization. The percentage of fever days was determined by dividing the amount of fever days by the total amount of days when a temperature was taken during the total hospitalization period.

Patients were divided into three groups: (1) no suspicion for bacterial ventriculitis; (2) clinical suspicion for bacterial ventriculitis, defined as initiation of empirical antibiotic treatment aimed specifically at bacterial ventriculitis, but negative CSF cultures; and (3) CSF culture-positive bacterial ventriculitis. Antibiotic regimen for bacterial ventriculitis consisted of ceftriaxone 2 g twice a day plus vancomycin 2 g twice a day, or ceftazidime 2 g three times a day plus vancomycin 2 g twice a day if an external CSF catheter was in place. Antibiotics were discontinued when the results of the CSF culture were negative (usually after 72 h) [6]. The antibiotic regimen was continued for two weeks if CSF cultures were positive.

To define a possible effect of SDD on the occurrence of bacterial ventriculitis and on the results of the CSF cultures, patients with suspected bacterial ventriculitis were divided into four groups: CSF culture-negative ventriculitis patients who receive SDD (group 1) or who do not receive SDD (group 2), and patients who have a CSF culture-positive bacterial ventriculitis who do (group 3) or do not receive (group 4) SDD.

### Statistical Methods

The obtained data were analyzed using Statistical Package for the Social Sciences 16.0 Software (IBM SPSS 19.0). Categorical variables were tested using a *χ* ^2^ or Fisher’s exact test. Continuous variables were tested with the Kolmogorov–Smirnov test for normal distribution. Normally distributed continuous variables are represented as a mean with a 95 % confidence interval (CI), and continuous variables which are not normally distributed are represented as a median with a 95 % confidence interval (CI). Normally distributed variables were tested with the Student’s *t* test (two group comparison) or a one-way ANOVA with Bonferroni post hoc analysis (multiple group comparison). Data that were not normally distributed were tested with the Mann–Whitney U test (two group comparison) or Kruskal–Wallis test (multiple group comparison). Values of *p* < 0.05 were considered as statistically significant.

## Results

### Participants

Between November 1, 2008, and October 31, 2010, 211 consecutive patients with an aneurysmal SAH were admitted. Two patients were excluded, leaving 209 patients for further analysis. All patients were initially admitted to the ICU. The clinical characteristics of 209 patients are presented in Table 1. Of the 149 coiled patients, 69 (46 %) had an external ventricular or lumbar catheter placed during the admission period, in contrast with 26 (90 %) of the 29 clipped patients.

**Table 1:** Clinical characteristics of 209 patients with an aneurysmal subarachnoid hemorrhage

Patients, *n*	209
Mean age, years (95 % CI)	54.8 (53.0–56.6)
Female, *n* (%)	130 (62)
Treatment, *n* (%)	
No aneurysm treatment	31 (15)
Coiling	149 (71)
Clipping	29 (14)
Decompressive craniectomy, *n* (%)	15 (7)
External ventricular catheter, *n* (%)	77 (37)
External lumbar catheter, *n* (%)	32 (15)
Cisternal catheter, *n* (%)	19 (9)
Lumbar puncture, *n* (%)	42 (25)
No CSF drainage, *n* (%)	108 (52)
WFNS grade, *n* (%)	
I–III	122 (58)
IV–V	87 (42)
Fisher grade, *n* (%)	
1–3	122 (58)
4	87 (42)

*WFNS* World Federation of Neurosurgical Societies, *CSF* cerebrospinal fluid

**Fig. 1:**
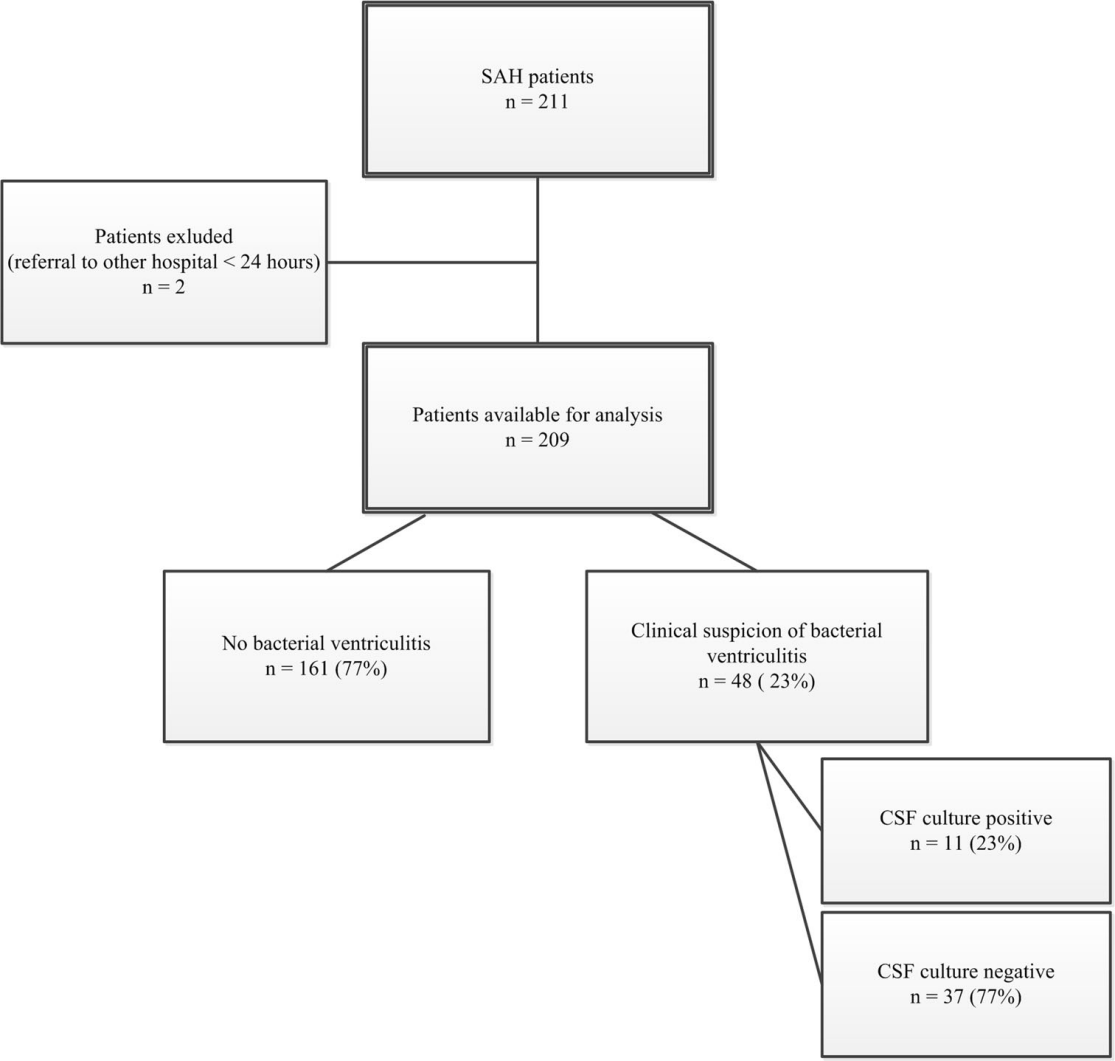
Flow chart regarding SAH patients with a clinical suspicion of bacterial ventriculitis. *CSF* cerebrospinal fluid

Forty-eight (23 %) patients were treated with antibiotics for a clinical suspicion of bacterial ventriculitis, and CSF cultures were positive in only 11 of these 48 patients (23 %; Fig. 1; Table 2).

**Table 2:** Clinical characteristics of three patient groups according to presence of bacterial ventriculitis

Characteristics			
Clinical	No suspected ventriculitis	Suspected ventriculitis	Suspected ventriculitis
CSF culture	Negative	Negative	Positive
Patients, *n*	161	37	11
Mean age, years (95 % CI)^†^	55.0 (52.9–57.1)	55.2 (51.4–59.0)	50.3 (40.8–59.7)
Female, *n* (%)	102 (63)	21 (57)	7 (64)
Median duration of hospitalization, days (95 % CI)^‡^	12.0 (11.0–13.0)^a^	23.0 (20.0–33.0)	34.0 (16.0–54.0)
WFNS grade, *n* (%)			
I–III	102 (63)^b^	17 (46)	3 (27)
IV–V	59 (37)^b^	20 (54)	8 (73)
Fisher grade, *n* (%)			
1–3	99 (62)	18 (49)	5 (46)
4	62 (38)	19 (51)	6 (54)
Treatment, *n* (%)			
No aneurysm treatment	30 (19)^b^	1 (3)	0 (0)
Coiling	111 (69)^b^	26 (70)	11 (100)
Clipping	19 (12)^b^	10 (27)	0 (0)
Decompressive craniectomy, *n* (%)	12 (8)	3 (8)	0 (0)
External ventricular catheter, *n* (%)	44 (27)^a^	24 (65)	9 (82)
External lumbar catheter, *n* (%)	13 (8)^a^	12 (32)	7 (64)
Cisternal catheter, *n* (%)	13 (8)^a^	6 (16)	0 (0)
Lumbar puncture, *n* (%)	50 (31)^a^	3 (5)	0 (0)
No CSF drainage, *n* (%)	98 (61)^a^	3 (8)	0 (0)
Median duration external CSF drainage, days (95 % CI)^‡^	6.0 (4.0–10.0)^a^	14.0 (8.0–17.0)^c^	19.0 (12.0–41.0)
Median duration of CSF drainage (days) of the external catheter preceding clinical suspicion of BM (95 % CI)^‡^	–	4.0 (4.0–6.0)^c^	14.0 (4.0–19.0)
SDD, *n* (%)	45 (28)^d^	20 (54)	5 (46)

*WFNS* World Federation of Neurosurgical Societies, *CSF* cerebrospinal fluid, *SDD* selective decontamination of the digestive tract

^†^One-way ANOVA

^‡^Kruskal–Wallis

^a^Significant difference (*p* < 0.05) between no suspected ventriculitis, and CSF culture-negative ventriculitis and CSF culture-positive bacterial ventriculitis

^b^Significant difference (*p* < 0.05) between no suspected ventriculitis and CSF culture-positive bacterial ventriculitis

^c^Significant difference (*p* < 0.05) between culture-negative ventriculitis and CSF culture-positive bacterial ventriculitis

^d^Significant difference (*p* < 0.05) between no ventriculitis and culture-negative bacterial ventriculitis

Bacteria cultured from CSF were *Staphylococcus epidermidis* (*n* = 8), *Enterococcus* species (*n* = 2), and Staphylococcus *aureus* (*n* = 1). One patient had two episodes of suspected ventriculitis during one hospitalization (14 days between episodes), but both episodes remained culture-negative.

Patients with bacterial ventriculitis were admitted in worse clinical condition, as reflected by higher WFNS grade at presentation, in comparison with patients without bacterial ventriculitis (median grade 4 vs. 2; *p* = 0.03). In total, 108 (52 %) patients received a CSF catheter: 75 patients (36 %) had primarily an external ventricular catheter placed, 16 (8 %) an external lumbar catheter and 17 (8 %) a cisternal catheter as the first form of CSF drainage. Seventy-nine patients (38 %) had only one drain (either ventricular or lumbar) placed during hospitalization, 21 had 2 external catheters (10 %) during admission, 7 patients (3 %) had 3 and 1 patient (1 %) even had 4 consecutive external catheters placed during hospitalization. Placement of external CSF catheters was associated with higher WFNS grades on admission (*p* < 0.001). All patients who had a culture-proven ventriculitis had one or more external ventricular catheters placed before they developed a bacterial ventriculitis. Of the 37 suspected patients in whom the CSF cultures came back negative 3 (8 %) never had an external CSF catheter. Among patients with an external CSF catheter placed, the number of days with a catheter in situ was associated with the diagnosis of suspected ventriculitis [median 14 days (8–17)] and culture-proven bacterial ventriculitis [median 19 days (12–41); Table 2]. The length of hospitalization was less with the diagnosis of suspected ventriculitis [median 23 days (20–33)] than with a culture-proven bacterial ventriculitis (median 34 days (16–54); Table 2).

### Clinical Symptoms and Chemical Analyses

The clinical symptoms and laboratory features in blood and CSF are presented in Table 3. Patients without ventriculitis showed significantly lower median temperatures and less fever days than both other groups (*p* < 0.05). There were no significant differences, however, in median temperatures and the median amount of fever days (both absolute and relative) between patients with CSF culture-positive bacterial ventriculitis and CSF culture-negative ventriculitis. The RBC count in CSF was significantly higher in patients with CSF culture-negative ventriculitis [102,900 cells/μL (33,600–177,000)] than in patients with CSF culture-positive bacterial ventriculitis [6300 cells/μL (1200–7,168,000); *p* = 0.01] (Table 3). Other clinical symptoms and laboratory features did not significantly differ between the groups.

**Table 3:** Clinical symptoms, cerebrospinal fluid and blood characteristics according to the presence of a positive CSF culture

Characteristics			
Clinical	No suspected ventriculitis	Suspected ventriculitis	Suspected ventriculitis
CSF culture	Negative	Negative	Positive
Patients, *n*	161	37	11
Nuchal rigidity, *n* (%)^‡^	–	11 (100)	6 (67)
Altered mental status, *n* (%)	–	9 (24)	4 (36)
Number of patients with >38.5 °C, *n* (%)	67 (42)^a^	32 (87)	10 (91)
Median *T*_max_ (95 % CI)^†,^*	37.5 (37.5–38.0)^a^	37.7 (37.5–38.0)	37.6 (37.3–38.3)
Median amount of fever days (95 % CI)^†,#^	0.0 (0.0–1.0)^a^	5.0 (2.0–7.0)	3.0 (1.0–11.0)
Median relative amount of fever days, % (95 % CI)^†,#^	0.0 (0.0–3.7)^a^	19.1 (11.1–32.4)	16.7 (4.0–47.1)
*CSF*			
Median RBC, cells/μL (95 % CI)^†^	–	102,900.0 (33,600.0–177,000.0)^b^	6300.0 (1200.0–7,168,000.0)
Median WBC, cells/μL (95 % CI)^†^	–	1722.0 (1191.0–2430.0)	3225.0 (489.0–15,595.0)
Median protein, g/L (95 % CI)^†^	–	1.0 (0.7–1.5)	1.2 (0.6–2.9)
Mean glucose, mmol/L (95 % CI)^¥^	–	3.4 (2.9–3.8)	2.7 (1.8–3.6)
*Serum*			
Median CRP, mg/L (95 % CI)^†^	–	33.3 (20.5–89.7)	40.9 (11.0–89.7)
Median WBC, 10E9/L (95 % CI)^¥^	–	12.7 (10.1–14.8)	15.1 (11.4–18.8)

*CSF* cerebrospinal fluid, *RBC* red blood cells, *WBC* white blood cells, *CRP* C-reactive protein

^†^Mann–Whitney U

^¥^Student *T* test

^‡^Nuchal rigidity was documented in 17 patients. Represented here is the percentage of patients in whom this was documented

* Measured until and including the day of clinical suspicion of bacterial ventriculitis

^#^Measured during the whole period of hospitalization

^a^Significant difference (*p* < 0.05) between no suspected ventriculitis and CSF culture-negative ventriculitis, and between no suspected ventriculitis and CSF culture-positive bacterial ventriculitis

^b^Significant difference (*p* = 0.01) between CSF culture-negative BM and CSF culture-positive BM

### Selective Decontamination of the Digestive Tract and Antibiotics

SDD was administered in 34 % of all patients. Patients treated for (suspected) bacterial ventriculitis received SDD more frequently than patients without bacterial ventriculitis; however, this difference was only significant between patients with culture-negative ventriculitis and patients without ventriculitis (*p* < 0.01). There was no significant difference between CSF culture-positive bacterial ventriculitis and CSF culture-negative ventriculitis (*p* = 0.74) (Table 2).

We found no significant differences in clinical characteristics, CSF features, or in duration of drainage, between CSF culture-negative ventriculitis with or without SDD and patients with CSF culture-positive bacterial ventriculitis with or without SDD. The CRP in blood was significantly (*p* = 0.01) lower [median 17.2 mg/L (1.6–40.9)] for patients with a CSF culture-positive bacterial ventriculitis using SDD than for patients with CSF culture-positive bacterial ventriculitis without SDD [median 69.1 mg/L (14.1–168.3)]. The WBC count in blood was significantly higher for patients with CSF culture-positive bacterial ventriculitis with SDD [mean 15.9 10E9/L (13.6–18.2)] compared to patients with CSF culture-positive bacterial ventriculitis without SDD [mean 14.7 10E9/L (10.7–18.7)] (*p* = 0.02). The CSF cultures of patients with CSF culture-positive bacterial ventriculitis yielded similar results for patients with or without SDD: Staphylococcus epidermidis in 4 of 6 patients without SDD, and in 4 of 5 patients with SDD.

Four of 11 (36 %) patients with culture-positive bacterial ventriculitis and 7 of 39 (18 %) patients with a culture-negative ventriculitis received antibiotics for another cause of infection before ventriculitis treatment (*p* = 0.23). None of the patients with CSF culture-positive bacterial ventriculitis and 12 of the 39 (31 %) patients with CSF culture-negative ventriculitis received antibiotics for another infection after discontinuation of antibiotic treatment (*p* = 0.05).

## Discussion

This study shows that almost a quarter of all patients admitted with an aneurysmal subarachnoid hemorrhage were treated with antibiotics for a clinical suspicion of bacterial ventriculitis, but that only in a small minority of these patients the CSF culture proved positive. A clinical suspicion of nosocomial bacterial ventriculitis should prompt a diagnostic workup and antimicrobial therapy. Our data show that clinical signs of ventriculitis are nonspecific and difficult to recognize in SAH patients who are sedated, who have recently undergone neurosurgery, or have a sterile inflammatory response in the CSF due to the SAH. Therefore, improvement of diagnostics for nosocomial bacterial ventriculitis in patients with aneurysmal SAH are needed to improve patients care and to reduce the administration of antibiotics, in order to lower the risk of antibiotic resistance development, and concomitantly to decrease health care costs.

The clinical symptoms of SAH, including headache, nuchal rigidity and altered mental status, closely resemble those of bacterial ventriculitis. These symptoms are thus non-specific and probably not useful to distinguish between both conditions. Fever, the most consistently reported feature in bacterial ventriculitis, occurs in 40 % of the patients after SAH with or without an infection [21–24]. The cell count in CSF may be helpful but has a low sensitivity and specificity [7]. The interpretation of white blood cells (WBC) in CSF is especially problematic in patients who develop bacterial ventriculitis after SAH as the presence of red blood cells (RBC) in CSF in itself causes an aseptic ventriculitis [9, 10, 13, 14]. The proposed cell index for external ventricular drainage-related ventriculitis in patients with intraventricular hemorrhage was not confirmed in subsequent series [13].

In the absence of better discriminative tests, treatment is usually initiated on first suspicion. Because nosocomial bacterial ventriculitis is associated with high mortality and morbidity [10, 14, 25, 26]. With this treatment strategy we aimed in this study that as few as possible bacterial ventriculitis patients were missed and as much as possible were treated, thus reducing the morbidity and mortality. Furthermore, the antibiotics were discontinued as soon as the cultures come back negative, which was normally after three days. Although the CSF culture (the most validated test for bacterial meningitis and ventriculitis) was used as gold standard for diagnosis, it is known that false negative tests may occur [21, 27, 28]. In the population under study, this could be further enhanced by the routine use of selective decontamination of the digestive tract in patients who were expected to be ventilator-dependent for at least two days. Patients who were treated for a (suspected) bacterial ventriculitis were more often in a worse clinical condition, hence the expectation to be ventilator-dependent. These poor condition patients are also the patients who are more prone to develop a bacterial ventriculitis [2]. Prophylactic antibiotics may mask a positive CSF culture leading to a negative CSF culture in patients with true bacterial ventriculitis. However, only one of the patients clinically suspected of bacterial ventriculitis in whom antibiotics were discontinued after a negative CSF culture experienced a second period of clinically suspected and culture-positive bacterial ventriculitis. In this patient, the first suspicion in retrospect was low. Fever reoccurred five days after discontinuation of the antibiotics for the first suspicion and was preceded by a drain blockage for which surgical revision was necessary.

CSF drainage procedures are common after a SAH and a longer duration of external drainage makes patients more susceptible to bacterial ventriculitis [7, 29, 30]. Not surprisingly, duration was significantly longer in patients with CSF culture-positive bacterial ventriculitis than in patients with CSF culture-negative bacterial ventriculitis. This is in concurrence with what is reported in the literature [7, 9, 15, 16].

Three patients had a clinical suspicion after a lumbar puncture as sole procedure and three did not even had any procedure (shunting, surgery or lumbar puncture) at all. Despite this fact, they did show clinical signs of bacterial ventriculitis, emphasizing the difficulty in clinical diagnosis.

We found no specific CSF features that distinguish in the early diagnosis of ventriculitis after SAH, although the CSF red blood cell (RBC) count was higher in patients with CSF culture-negative bacterial ventriculitis than in patients with CSF culture-positive bacterial ventriculitis. The influx of RBCs in the CSF after SAH causes CSF disturbances, and these have been shown to have a poor discriminatory value [10, 13, 31–33]. Other substrates, such as CSF lactate, cytokine levels, and serum procalcitonin, are also disturbed after SAH. Although procalcitonin has been shown to be able to discriminate between a systemic inflammatory response syndrome and a systemic infection [34], the value to differentiate between aseptic and bacterial ventriculitis of this and other substrates is limited [33–38]. Polymerase chain reaction of the CSF for the detection of bacterial pathogens has been shown to have a low sensitivity in patients with an external catheter-related bacterial ventriculitis and patients with aseptic ventriculitis after surgery, but assays are getting more sensitive [39–42].

*S. epidermidis* was the most common pathogen found in the CSF cultures, as in other studies on external catheter-related bacterial ventriculitis [7, 9, 10, 14–16, 43]. However, the frequency of *S. aureus* as causative pathogen was lower than described in the literature, possibly the result of selective SDD which is aimed at reducing infection with *S. Aureus* [19, 44].

Limitations of this study are its retrospective nature and the small sample of patients with CSF culture-positive bacterial ventriculitis. Data were collected from clinical records, and underreporting of clinical suspicion and features may have biased the results. However, patients were only categorized as CSF culture-negative ventriculitis if antibiotics were started for this suspicion, which is a well-defined criterion with a computerized medication prescription system used in our facility. The choice of antibiotics is also quite specific and thus readily traceable to a tentative diagnosis of bacterial ventriculitis.

In this retrospective series of SAH patients, nosocomial bacterial ventriculitis is often suspected but confirmed by culture in a minority of cases. The clinical signs of ventriculitis are nonspecific and difficult to recognize in SAH patients. Improvement of diagnostics for nosocomial bacterial ventriculitis in patients with aneurysmal SAH is needed.
